# Evaluation of Four Different Automated Activity Monitoring Systems to Identify Anovulatory Cows in Early Lactation

**DOI:** 10.3390/ani14213145

**Published:** 2024-11-02

**Authors:** Lukas Frieder Bretzinger, Marvin Hölper, Christie Marie Tippenhauer, Jan-Lukas Plenio, Augusto Madureira, Wolfgang Heuwieser, Stefan Borchardt

**Affiliations:** 1 Farm Animal Clinic, Unit for Reproduction Medicine and Udder Health, Division for Ruminants and Camelids, Faculty of Veterinary Medicine, Freie Universitaet Berlin, Koenigsweg 65, 14163 Berlin, Germany; l.bretzinger@fu-berlin.de (L.F.B.);; 2 Institute for Veterinary Epidemiology and Biostatistics, Freie Universitaet Berlin, Koenigsweg 67, 14163 Berlin, Germany; 3 Department of Animal Science, Michigan State University, East Lansing, MI 48824, USA; 4 Cornell University College of Veterinary Medicine, 240 Farrier Rd., Ithaca, NY 14853, USA

**Keywords:** anovulation, estrous expression, automated activity monitoring, reproductive performance

## Abstract

The objective of this study was to evaluate four different automated activity monitoring (AAM) systems to identify anovulatory cows in early lactation. A total of 852 lactating Holstein cows (221 primiparous and 631 multiparous cows) from four commercial dairy herds were enrolled. On each farm, cows were equipped with a respective AAM system (SB: Smartbow; HT: Heatime; DP: Delpro; and CM: CowManager). Each cow was sampled three times within the voluntary waiting period (VWP) in a two-week interval to detect the blood progesterone (P4) concentration. Cows were classified based on the concentration of P4 as follows: (1) none of the three blood P4 concentrations exceeded 1.0 ng/mL (anovulatory); (2) at least one of the three blood P4 concentrations was above 1.0 ng/mL (ovulatory). Cows were classified based on estrus alerts as follows: (1) no estrus alert was detected by an AAM system from 7 to 60 DIM (anestrus); (2) at least one estrus alert was detected by an AAM system from 7 to 60 DIM (estrus). Accuracy, sensitivity, specificity, positive predictive value, and negative predictive value were calculated for each AAM system for anovulatory cows [(SB: 77.6%; 26.8%; 89.3%; 36.7%; and 84.1%); (HT: 79.2%; 63.6%; 83.6%; 52.8%; and 88.9%); (DP: 47.2%; 78.8%; 41.5%; 19.5%; and 91.6%); (CM: 80.5%; 23.7%; 92.7%; 39.1%; and 85.3%)].

## Simple Summary

Data collected by automated activity monitoring (AAM) systems during the voluntary waiting period (VWP) can help detect cows with poor fertility outcomes. The objective of this study was to evaluate four different AAM systems to identify anovulatory cows from 7 to 60 days in milk. Automated systems differed in their test characteristics to detect anovulatory cows. Anovulatory cows within the VWP had an inferior reproductive performance compared with ovulatory cows. Cows expressing estrus during the VWP had improved reproduction performance compared with cows that did not show estrus.

## 1. Introduction

Activity data from automated activity monitoring (AAM) systems within the voluntary waiting period (VWP) can be used to identify cows that have poor reproductive performance. Cows that showed signs of estrus detected by an AAM system one or multiple times from 7 to 40 days in milk (DIM) [1], 21 to 49 DIM [2], or 7 to 60 DIM [3] had superior reproductive performance compared to cows without an estrus event during the VWP. Cows with an estrus event detected by an AAM system within the VWP were more likely to get bred via artificial insemination (AI) at the first estrus event after the VWP than to receive timed AI (TAI), had greater pregnancy per AI (P/AI), and were more likely to get pregnant by 150 DIM [2] or 200 DIM [1,3]. Tailoring interventions or treatments to subgroups of cows in a herd that share specific biological characteristics or expected performance can be a beneficial management approach [4]. Data from AAM systems within the VWP can support early decision making [4].

Several AAM systems used on farms have been validated, for estrus detection, in the past [5,6,7,8]. To determine the accuracy of an AAM system, estrus events identified by the system are compared with a gold standard such as visual estrus observation, transrectal ultrasound (US) examination of the ovaries, blood or milk progesterone (P4) concentrations, or a combination of these. Sensitivity and specificity differed for the AAM systems: HT: 85% and 86.9% [5]; CM: 100% and 98.6% [6]; SB: 89.6% and 86.7% [7]; and DP: 82.5% and 88.8% [8].

All of these studies have been performed using cows after the VWP ([8]: 35 DIM; [5]: 46 DIM; [7]: 47 DIM; and [6]: 57 DIM). So far, AAM systems have not been validated for estrus detection within the VWP using blood P4 concentration as a gold standard. The detection of estrus events within the VWP might be advantageous for reproductive management but more challenging for AAM systems as the first ovulation after parturition is often associated with weak estrus behavior [1,9].

Therefore, the objective of this study was to evaluate four different AAM systems to identify anovulatory cows from 7 to 60 DIM. Our hypothesis was that AAM systems can be used to detect anovulation within early lactation to facilitate targeted reproductive management. Specifically, we did not expect a major difference in test characteristics (i.e., sensitivity and specificity) among the different AAM systems.

## 2. Materials and Methods

### 2.1. Animals, Housing, and Management

This prospective, longitudinal observational study included blood P4 concentrations and AAM data from 7 to 60 DIM from 852 lactating Holstein cows (221 primiparous and 631 multiparous cows) from four commercial dairy farms (Table 1) in northeastern Germany, calving from July 2021 to December 2022. Inclusion criteria were a farm size of more than 500 cows and the use of an AAM system. Cows were excluded after enrollment in this study if they had less than 95% usable AAM data from 7 to 60 DIM (*n* = 32), and if they were culled or left the farm before 60 DIM (*n* = 83). Farm size ranged from approximately 615 to 1003 cows per farm (Table 1). Cows were housed in freestall barns, had *ad libitum* access to feed and water, and were fed twice daily a total mixed ration (TMR) diet consisting of corn silage and grass silage as forage with a corn and canola meal-based concentrate. The TMR was formulated to meet or exceed the dietary requirements for lactating dairy cows [10]. Cows were milked twice (farms 3 and 4) or three times (farms 1 and 2) daily in a rotary milking parlor with a milk yield ranging from 10,008 to 11,598 kg per 305 d (Table 1). The voluntary waiting period was 42 d (farms 1 and 3) or 60 d (farms 2 and 4). Lactating cows were inseminated based on estrus detection following an alert of the AAM system after the VWP of the respective farm. Estrus was verified by the AI technician via transrectal palpation of a highly contractile uterus, visualization of clear, stringy vaginal discharge, or both. If cows were confirmed in estrus, AI was conducted on the same day. Inseminations were performed with conventional semen. Cows showing signs of estrus at any time after an AI (e.g., first AI) received further AI services. Pregnancy diagnosis was performed by the veterinarian of the respective farm at 42 d after AI via US. All cow-related events and procedures (e.g., estrus, AI, treatments, and pregnancy outcomes) were entered into the farm management software (herdeplus; dsp-Agrosoft GmbH, Ketzin, Germany) by farm personnel.

### 2.2. Experimental Blood Collection and Scoring

On each farm, a work list of cows to score was generated in the farm management software. Each cow included in the experiment was blood-sampled three times to detect the blood P4 concentration within the first 60 DIM. The first sample (P1) was taken between 14 and 27 DIM, the second (P2) two weeks after the first sample, between 28 and 41 DIM, and the third sample (P3) two weeks after the second sample, between 42 and 55 DIM (±1 d). The overall DIM averages for sampling were 20.7 ± 4.1 (P1), 34.7 ± 4.1 (P2), and 48.8 ± 4.1 (P3). Every time a cow was sampled, body condition and locomotion were scored as well. For the body condition score (BCS), a 5-point scale was used with 0.25 increments (1 = thin to 5 = fat) [11]. Cows were defined with BCS loss when the BCS difference between the first and the last time of scoring (P1 vs. P3) had a negative value. Locomotion score (LMS) was evaluated using a 5-point scale (1 = non-lame to 5 = severe lameness) [12]. Score 1 is a cow that stands and walks normally with a level-back posture and a normal gait. Score 2 is a cow that stands with a level-back posture but develops an arched-back posture while walking but with a normal gait. Score 3 is a cow with an arched-back posture evident both while standing and walking. Score 4 is a cow with an arched-back posture always evident and an abnormal gait favoring one limb. Score 5 is a cow that demonstrates an inability or extreme reluctance to bear weight on one or more limbs. Cows were defined as lame when they were scored 3, 4, or 5 at least once (P1/P2/P3). All visual scorings were performed by the same person (LB). Blood samples were collected by venipuncture of the coccygeal vessels, using a 20-gauge, 1.5-inch hypodermic needle (Vacuette, Greiner Bio-One, Monroe, NC, USA). Blood was extracted into a sterile, plastic, evacuated serum collection tube with no anticoagulant (8 mL, Vacuette, Greiner Bio-One, Monroe, NC, USA) and centrifuged after collection on the dairy farm at 4500× *g* for 10 min at approximately 20 °C, and then stored on ice for transport to the laboratory of the Farm Animal Clinic, Division for Ruminants and Camelids, Unit for Reproduction Medicine and Udder Health. At the clinic, serum was transferred into sterile vials (2 mL, Cryovial, Simport, Beloeil, QC, Canada) and stored at −18 °C until shipment to a commercial laboratory (SYNLAB.vet, accreditation number ISO 17025, Berlin, Germany). Serum P4 concentrations were determined using an enzyme-labeled chemiluminescent competitive immunoassay (Immulite Progesterone Enzym, Siemens Healthcare). Intra- and inter-assay coefficients of variation for 20 assays for repeated samples averaged 8.61 ± 0.32% and 8.05 ± 0.51%, respectively. The lower limit of detection was 0.2 ng/mL.

### 2.3. Automated Activity Monitors

All cows were equipped with an AAM system (SB: Smartbow, Zoetis Inc., New Jersey, USA; HT: Heatime, SCR Engineers Ltd., Netanya, Israel; DP: Delpro, Tumba, Sweden, AM 2, DeLaval; and CM: CowManager SensOor, Agis Automatisering, Harmelen, Netherlands) before calving (Table 1). Activity data of each individual cow were recorded in real time (SB, 1 min periods; HT, 2 h periods; and DP and CM, 1 h periods) by a wireless receiver box and transmitted to the on-farm computer, where the respective accelerometer software was installed (Table 1). These AAM systems have been validated for estrus detection previously (SB [13]; HT [14]; DP [8]; and CM [6]).

For each cow, a file from the AAM system was generated in the respective AAM system software on the farm computer (Table 1) including cow ID, time of estrus alert (dd.mm.jjjj, hh:mm) (SB), and additionally, activity level (DP), DIM at estrus alert, duration of each estrus event (CM), and raw activity data (HT). This file was exported in XLSX format from EXCEL (Office 2016, Microsoft Deutschland Ltd., Munich, Germany). Files from the SCR system were exported using DataFlow II on a weekly basis. A software tool (BovHEAT v1.2.0) [15], written in the open-source Python programming language (Python Software Foundation, v3.12.0), was used to process all XLSX files and generate a single result report XLSX file. Files from the Delpro system were exported using Delpro Farm Management. A cow was considered to have an estrus event when the activity level estimated on a 3-point scale reached the highest level. For the CowManager system, the CowManager Control Center was used. A cow was considered to have an estrus event when the activity level (potential/suspicious/in heat) was displayed as “in heat”. For the Smartbow system, the Smartbow software was used to depict the data of each cow, and all data were exported manually. The processed and cleaned report included the following information for each cow: cow ID, lactation number, calving date, number of estrus events, DIM at estrus event, time of estrus event (dd.mm.jjjj, hh:mm), and duration of each estrus event.

For all four AAM systems, a period from 7 to 60 DIM was chosen. Activity data from 0 to 6 DIM were excluded because the AAM system needed time to generate baseline activity data after calving. Additionally, a lot of false positive alarms were generated by all AAM systems in the first 6 DIM. This might be due to a higher activity caused by the calving event and group changes.

Based on the P4 samples concentration, cows were classified as follows: (1) none of the three blood P4 concentrations exceeded 1.0 ng/mL (anovulatory; (2) at least one of the three blood P4 concentrations was above 1.0 ng/mL (ovulatory). Based on estrus alerts detected by an AAM system from 7 to 60 DIM, cows were classified as follows: (1) no estrus alert (anestrus); and (2) at least one estrus alert (estrus).

### 2.4. Statistical Analyses

Cow ID, parity, and calving date were obtained through the on-farm computer software herdePlus. For each AAM system and farm, all data were transferred to EXCEL and merged with the respective activity data report and the results of the blood P4 samples via ACESS (Office 2016, Microsoft Deutschland Ltd., Munich, Germany). All statistical analyses were performed using SPSS for Windows (version 28.0, SPSS Inc., IBM, New York, USA).

Accuracy, sensitivity, specificity, positive predictive value, and negative predictive value were calculated for each AAM system to detect a cow that ovulated within 60 DIM. An anovulatory cow (blood P4 concentration did not exceed 1.0 ng/mL) with no estrus alert from 7 to 60 DIM was defined as a true positive (TP). An ovulatory cow (at least one blood P4 concentration exceeded 1.0 ng/mL) with at least one estrus alert from 7 to 60 DIM was considered to be a true negative (TN). A false positive (FP) was defined as no estrus alert from 7 to 60 DIM in an ovulatory cow. A false negative (FN) was defined as at least one estrus alert from 7 to 60 DIM in an anovulatory cow. Accuracy was calculated by dividing the TP by all positives [TP/(TP + FP)]. Sensitivity was calculated by dividing the TP by the sum of the TP and FN [TP/(TP + FN)], whereas specificity was determined by dividing the TN by the sum of the FP and TN [TN/(FP + TN)]. The positive predictive value was determined by dividing the TP by all test positives [TP/(TP + FP)]. The negative predictive value was determined by dividing the TN by all test negatives [TN/(TN + FN)].

To evaluate the association between estrous expression detected by an AAM system indicative of resumption of cyclicity within 60 DIM and P/AI at first postpartum AI, two separate logistic models were built using the GENLINMIXED procedure of SPSS. Herd was considered a random effect. Cow was nested within farm. Model building was conducted as recommended by Dohoo et al. [16], where each parameter was first analyzed separately in an univariable model using the GENLINMIXED procedures as described above. Only parameters resulting in univariable models with *p* ≤ 0.10 were included in the final mixed models. Selection of the model that best fits the data was performed using a backward stepwise elimination procedure by removing all variables with *p* > 0.10 from the model. The initial models included the following explanatory variables as fixed effects: parity (primiparous vs. multiparous), BCS loss (cows losing BCS vs. cows maintaining or gaining BCS), lameness (lame vs. non-lame), and estrous expression from 7 to 60 DIM (anestrus vs. estrus) or resumption of cyclicity within 60 DIM (anovulatory vs. ovulatory).

Cox proportional hazards were used to model the time-to-event outcomes (i.e., time to first AI, time to pregnancy). Cows were excluded from the final analysis if they were culled before first insemination, pregnancy diagnosis, or the end of the observation period. The variable parity, season of calving, lameness, BCS loss, farm, and estrous expression from 7 to 60 DIM (anestrus vs. estrus) or resumption of cyclicity within 60 DIM (anovulatory vs. ovulatory) were tested as risk factors. The proportional hazard assumption was checked using Schoenfeld residuals.

The frequency distribution of estrus events from 7 to 60 DIM and ovulatory status was tested for parity, lameness, and BCS loss using a Chi-Square test.

To account for multiple comparisons, the *p*-value was adjusted using a Bonferroni correction. Variables were declared to be significant when *p* ≤ 0.05. A statistical tendency was declared when *p* was between 0.05 and ≤0.10.

## 3. Results

### 3.1. Descriptive Statistics

Overall, 852 cows were included in the final statistical analyses. Out of these, 18.3% (156/852) were anovulatory and 28.1% (239/852) were anestrous cows (Table 2). Parity (*p* = 0.011), lameness (*p* = 0.001), and BCS loss (*p* = 0.022) were associated with the frequency of anovulatory cows within 60 DIM. The proportion of anovulatory cows was greater in primiparous (23.9%; 53/221) compared to multiparous cows (16.3%; 103/631). A greater proportion of lame cows (32.0%; 56/175) was anovulatory compared with non-lame cows (14.8%; 100/677). Cows that lost BCS (20.4%; 119/584) had a greater hazard of being anovulatory compared to cows that maintained or gained BCS (13.8%; 37/268). The cumulative prevalence for anovulatory cows was 68.8, 36.4, and 18.3% at weeks 3, 5, and 7, respectively.

Lameness (*p* = 0.021) and BCS loss (*p* = 0.005) were associated with the number of estrus events from 7 to 60 DIM. A greater proportion of lame cows (35.4%; 113/175) had no estrus event from 7 to 60 DIM compared to non-lame cows (26.1%; 177/677). A greater proportion of cows that lost BCS (31.0%; 181/584) had no estrus event from 7 to 60 DIM compared to cows that maintained or gained BCS (21.6%; 58/268). Parity had no effect (*p* = 0.164) on the frequency of estrus events from 7 to 60 DIM.

### 3.2. Characteristics of the First Postpartum Estrus Event

**Figure 1:**
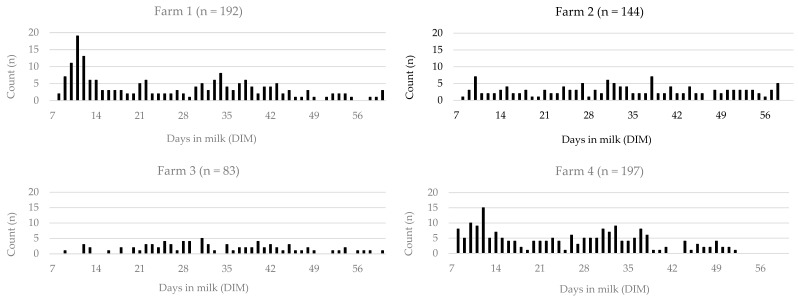
Frequency distribution of days in milk (DIM) at first estrus event for 617 cows from 4 farms. Cows were equipped with an automated activity monitoring (AAM) system (farm 1: Smartbow, Zoetis Inc.; farm 2: Heatime, SCR Engineers Ltd.; farm 3: Delpro, AM 2, DeLaval; and farm 4: CowManager SensOor, Agis Automatisering). An estrus event was defined as an estrus alert of the respective AAM system from 7 to 60 DIM.

Frequency distribution of DIM at the first estrus event for 617 cows [farm 1 (*n* = 193 cows); farm 2 (*n* = 144 cows); farm 3 (*n* = 83 cows); and farm 4 (*n* = 197 cows)] detected by the respective AAM system is shown in Figure 1. Overall, the median DIM at the first estrus event was 29 (farm 1: 25 DIM; farm 2: 32.5 DIM; farm 3: 32 DIM; and farm 4: 26 DIM). Days in milk at first postpartum estrus event were affected by farm (*p* < 0.001), parity (*p* = 0.009), cyclicity within VWP (*p* < 0.001), season of calving (*p* < 0.001), and lameness (*p* = 0.033). Body condition loss was not associated with DIM at the first postpartum estrus event (*p* = 0.866). The mean DIM at the first estrus event was 26.6 ± 1.03 DIM, 33.0 ± 1.21 DIM, 33.3 ± 1.33 DIM, and 25.3 ± 0.88 DIM for farms 1, 2, 3, and 4, respectively. Multiparous cows had their first estrus event earlier (27.7 ± 0.65 DIM) than primiparous cows (30.9 ± 1.1 DIM). Cows that were ovulatory within the VWP had their first estrus earlier (27.7 ± 0.59 DIM) compared with cows that were anovulatory (34.2 ± 1.59 DIM). Cows with lameness had their first estrus event later (31.1 ± 1.3 DIM) than non-lame cows (28.0 ± 0.62 DIM). Cows calving in winter had a longer interval from calving to the first estrus event (34.9 ± 1.31 DIM) compared with summer (27.6 ± 1.07 DIM) and autumn (25.7 ± 0.78 DIM). The interval from calving to the first estrus event did not differ between winter and spring (34.0 ± 1.71 DIM).

### 3.3. Validation of the Respective AAM System

Accuracy, sensitivity, specificity, positive predictive value, and negative predictive value were calculated for each AAM system to detect a cow that did not ovulate within 60 DIM. The results are summarized in Table 3 and Table 4. The AAM system CM had the highest accuracy (80.5%), followed by HT (79.2%), SB (77.6%), and DP (47.2%). For accuracy, DP differed from the other three AAM systems. DP had the highest sensitivity (78.8%), followed by HT (63.6%), SB (26.8%), and CM (23.7%). For sensitivity, the neck-attached AAM systems HT and DP differed from the ear-attached systems SB and CM. CM had the highest specificity (92.7%), followed by SB (89.3%), HT (83.6%), and DP (41.5%). For specificity, DP differed from the other three AAM systems. HT had the highest positive predictive value (52.8%), followed by CM (39.1%), SB (36.7%) and DP (19.5%). DP had the highest negative predictive value (91.6%), followed by CM (85.3%), SB (84.1%), and HT (88.9%).

### 3.4. Pregnancy per AI at First AI

Overall P/AI was 38.6% (311/810). Pregnancy per AI at first postpartum AI was affected by parity (*p* = 0.002). Primiparous cows (46.0 ± 3.1%) had greater P/AI compared with multiparous cows (32.4 ± 1.8%: *p* = 0.002). There tended to be an association between P/AI and estrous expression from 7 to 60 DIM (*p* = 0.074), as cows with estrous expression (42.9 ± 2.0%) tended to have greater P/AI compared with cows without estrous expression (35.2 ± 3.2%).

Resumption of cyclicity within 60 DIM (i.e., anovulatory vs. ovulatory) was not associated with P/AI at first service (*p* = 0.598).

### 3.5. Time to First AI

**Figure 2:**
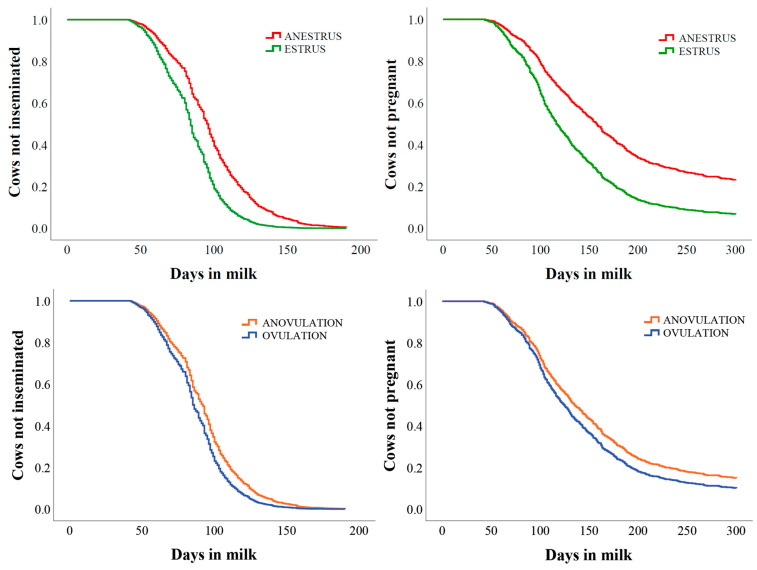
Kaplan–Meier survival analyses illustrating the association of estrous expression from 7 to 60 DIM with time to first insemination (**top left**) and time to pregnancy (**top right**) in 852 cows. Kaplan–Meier survival analyses illustrating the association of ovulation and anovulation within 60 DIM with time to first insemination (**bottom left**) and with time to pregnancy (**bottom right**) in 852 cows. Cows were equipped with an automated activity monitoring (AAM) system (farm 1: Smartbow, Zoetis Inc.; farm 2: Heatime, SCR Engineers Ltd.; farm 3: Delpro, AM 2, DeLaval; and farm 4: CowManager SensOor, Agis Automatisering). An estrus event was defined as an estrus alert of the respective AAM system from 7 to 60 DIM. Cows were classified as (1) no estrus alert from 7 to 60 DIM by the respective AAM system (anestrus; red line; *n* = 239); (2) at least one estrus alert from 7 to 60 DIM by the respective AAM system (estrus; green line; *n* = 613); (3) none of the three blood progesterone concentrations exceeded 1.0 ng/mL (anovulatory; orange line; *n* = 156); and (4) at least one of the three blood progesterone concentrations was above 1.0 ng/mL (ovulatory; blue line; *n* = 696). The first blood progesterone sample (P1) was taken between 14 and 27 DIM, the second (P2) two weeks after the first sample, between 28 and 42 DIM, and the third sample (P3) two weeks after the second sample, between 43 and 56 DIM.

Median DIM to first AI were 93 and 86 for anovulatory and ovulatory cows as shown in Figure 2. Time to first AI was affected by parity (*p* < 0.001), season of calving (*p* = 0.003), estrous expression from 7 to 60 DIM (*p* = 0.012), lameness (*p* = 0.032), and farm (*p* < 0.001). Ovulatory cows had an increased hazard [hazard ratio (HR) = 1.30 (1.09–1.56); *p* = 0.004] of being inseminated within 200 DIM compared to anovulatory cows. Multiparous cows had a reduced hazard [HR = 0.64 (0.54–0.75); *p* < 0.001] of being inseminated within 200 DIM compared with primiparous cows. Cows calving in summer [HR = 0.27 (0.21–0.35); *p* < 0.001] and autumn [HR = 0.34 (0.28–0.42); *p* < 0.001] had a reduced hazard of insemination within 200 DIM compared to spring. Cows calving in winter tended to have an increased hazard [HR = 1.22 (0.98–1.52); *p* = 0.081] of being inseminated by 200 DIM. Compared to farm 4, farm 1 [HR = 0.69 (0.57–0.84); *p* < 0.001] had a reduced, and farms 2 [HR = 8.56 (6.77–10.80); *p* < 0.001] and 3 [HR = 3.25 (2.66–3.96); *p* < 0.001] had an increased hazard of being inseminated by 200 DIM. Lame cows had a reduced hazard [HR = 0.81 (0.68–0.96); *p* = 0.015] of being inseminated within 200 d compared to non-lame animals.

Cows with an estrus event from 7 to 60 DIM had an increased hazard [hazard ratio (HR) = 1.80 (1.51–2.15); *p* < 0.001] of being inseminated by 200 DIM. The median DIM at first insemination results were 95 and 84 for anestrous and estrous cows. Ovulatory cows had an increased hazard [HR = 1.30 (1.09–1.56); *p* = 0.004] of being inseminated within 200 DIM compared to anovulatory cows. The median DIM at first insemination results were 93 and 86 for anovulatory and ovulatory cows, respectively. Cows with estrous expression from 7 to 60 DIM had an increased hazard [HR = 1.84 (1.52–2.24); *p* < 0.001] of becoming pregnant by 300 DIM compared with anestrous cows. The median DIM to pregnancy results were 143 and 121 for anestrous and estrous cows. Ovulatory cows [HR = 1.21 (0.99–1.47); *p* = 0.068] had an increased tendency to become pregnant within 300 DIM compared to anovulatory cows. The DIM at pregnancy results were 124 and 118 for anovulatory and ovulatory cows, respectively.

The median DIM to first insemination results were 84 and 95 for cows with and without an estrus event detected by an AAM system from 7 to 60 DIM as shown in Figure 2. Cows with an estrus event from 7 to 60 DIM had an increased hazard [HR = 1.80 (1.51–2.15); *p* < 0.001] of being inseminated by 200 DIM.

### 3.6. Time to Pregnancy

The median DIM to pregnancy were 124 and 118 for anovulatory and ovulatory cows, respectively, as shown in Figure 2. Time to pregnancy was affected by parity (*p* < 0.001), farm (*p* < 0.001), lameness (*p* < 0.001), and estrous expression from 7 to 60 DIM (*p* < 0.001). Ovulatory cows [HR = 1.21 (0.99–1.47); *p* = 0.068] had an increased tendency to become pregnant within 300 DIM. Multiparous cows had a reduced hazard [HR = 0.62 (0.53–0.74); *p* < 0.001] of becoming pregnant within 300 DIM compared with primiparous cows. Compared to farm 4, farm 1 [HR = 0.72 (0.59–0.84); *p* = 0.002] had a reduced hazard of becoming pregnant by 300 DIM. Lame cows had a reduced hazard [HR = 0.68 (0.56–0.83); *p* < 0.001] of becoming pregnant by 300 DIM compared to non-lame animals.

The median DIM to pregnancy results were 121 and 143 for cows with and without an estrus event detected by an AAM system from 7 to 60 DIM as shown in Figure 2. Cows with estrous expression from 7 to 60 DIM had an increased hazard [HR = 1.84 (1.52–2.24); *p* < 0.001] of becoming pregnant by 300 DIM compared with ANESTRUS cows.

## 4. Discussion

The objective of this study was to evaluate the performance of four AAM systems to identify anovulatory cows from 7 to 60 DIM. This is the first study that has evaluated different AAM systems to identify anovulatory cows in early lactation. Our results provide evidence that test characteristics vary between AAM systems. In addition, we were able to identify lameness and BCS loss in early lactation as important risk factors for anovulatory cows and the probability of an estrus event within the VWP. Moreover, we compared the reproductive performance of cows based on the ability of the AAM system to detect anovulatory cows and the expression of estrus within the VWP. Ovulation and estrous expression within the VWP were associated with improved reproductive performance. Particularly, cows with an estrus event within the VWP had an increased hazard of being inseminated by 200 DIM and becoming pregnant within 300 DIM.

### 4.1. Prevalence of Anovulation

The overall prevalence of anovulatory cows was 18.3% in our study. Within herds, the prevalence ranged from 15.3 to 22.3%. Our results are in agreement with other studies that evaluated the prevalence of anovulation in early lactation ([17]: 19.5%, 18 herds, milk P4 at 46 and 60 DIM; [18]: 23.3%, 17 herds, blood P4 or US between 50 and 65 DIM). The prevalence of anovulation decreased within the VWP. The cumulative prevalence of anovulatory cows was 68.8, 36.4, and 18.3% in weeks 3, 5, and 7, respectively. This is in agreement with a previous study [19] in which the cumulative prevalence of anovulation was 72, 44, and 17% in weeks 3, 5, and 7, respectively. Parity was associated with the prevalence of anovulatory cows (primiparous cows: 23.9% vs. multiparous cows: 16.3%), which has been shown previously ([18]: 29.6% for 2318 primiparous cows and 19.1% for 3500 multiparous cows).

### 4.2. Prevalence of Anestrus

The overall prevalence of anestrous cows was 28.1% in our study. Within herds, prevalence ranged from 10.5 to 61.6%. Each herd used, however, a different AAM system. Studies that also used an AAM system to evaluate the prevalence of anestrus observed a greater number of anestrous cows than in our study ([1]: 52.1%, 5 herds, Heatime SCR, 7 to 40 DIM; [20]: 52.7%, 1 herd, within 62 DIM, Heatime SCR), whereas other studies had a lower prevalence for anestrous cows ([3]: 20.8%, 1 herd, Nedap Smartneck, 7 to 60 DIM). Interestingly the prevalence of anestrus differed compared to the prevalence of anovulatory cows. This discrepancy is due to the different accuracy of AAM systems to identify anovulatory cows. The AAM systems seem to have trouble identifying anovulatory cows early in lactation since the first ovulation is often not accompanied by estrus activity [9]. It is believed that elevated estradiol levels around the time of parturition lead to a resistant state against the estrogens present during the first postpartum ovulation. Nevertheless, P4 secreted from the corpus luteum (CL) after the first ovulation appears to support estrous expression in the subsequent ovulatory cycle [21].

### 4.3. Risk Factors for Anovulation and Anestrus

Several factors have been associated with anovulation in the VWP such as parity, digestive disorders, ketosis, displaced abomasum, body weight loss (>28 kg), negative energy balance, higher blood concentrations of non-esterified fatty acids or haptoglobin [19], dystocia, twins [17,22,23], and extensive longissimus dorsi muscle loss (>8 mm) postpartum [24]. Inflammatory processes in the reproductive tract seem to have a major impact on anovulation within the VWP [25]. Therefore, metritis [22,23], cytological endometritis [19], and purulent vaginal discharge [25] were considered important risk factors. In a study from our group, we found factors associated with anestrus in the VWP to be stillbirth, retained placenta, metritis, and subclinical ketosis [3].

### 4.4. Test Performance of the AAM Systems Used

There was considerable variation in the ability to identify anovulatory cows among the four AAM systems. One has to keep in mind, that a direct comparison among the AAM systems is difficult as they were not mounted on the same cows on the same farm. Interestingly, the ear-attached AAM systems (SB and CM) had lower sensitivities compared to the neck-mounted AAM systems (HT and DP). Two previous studies also assessed the performance of a neck-mounted [5] or ear-attached [7] AAM system. However, in these studies, cows were synchronized with GnRH followed by PGF 7 d later. Their results showed that 71% [5] and 84% [7] of cows were detected in estrus by the AAM system and 95% of cows showing estrus ovulated within 7 d after induction of luteolysis. Of the cows not detected in estrus by the AAM system, 35% [5] and 62% [7] ovulated within 7 d after induction of luteolysis. The results of these two studies suggest that the ability of an AAM system to detect ovulation in cows past the VWP is different compared to cows within the VWP. In a study from Peixoto et al. [26], a neck-mounted AAM system was used to monitor postpartum cyclicity based on the diagnosis of a CL (≥20 mm) via US at 29 and 43 DIM ± 3. They concluded that this AAM system was not able to determine cyclicity in cows based on estrus events up to 43 ± 3 DIM. Compared with the performance of HT (79.2%) in our study, the authors described a reduced accuracy (59%). A direct comparison of these studies, however, is difficult as observation times (43 vs. 60 DIM) and reference methods for cyclicity (US vs. P4 concentration) differed. Using US as a gold standard to determine cyclicity is a study limitation. This might introduce some bias as cows with an active CL (P4 ≥ 1 ng/mL) are misclassified. Sensitivity ranged from 89.4% to 91.2% and specificity ranged from 39.8% to 80% in three studies that evaluated US to identify cows with an active CL using blood P4 [27,28,29].

### 4.5. Reproductive Performance of Cows with Anovulation and Anestrus

Ovulatory cows had an increased hazard of being inseminated within 200 DIM compared with anovulatory cows. The median DIM at first insemination results were 93 and 86 for anovulatory and ovulatory cows, respectively. This corresponds with two studies in which anovulatory cows had a reduced likelihood of being inseminated [17,30]. In the first study [17], using milk P4 profiles for the detection of anestrus within the VWP in herds with minimum hormonal interventions, the median DIM at first insemination results were 80 and 72 for anovulatory and ovulatory cows, respectively. In the second study [30], a Presynch–Ovsynch protocol with insemination after estrus detection was used to facilitate the first postpartum TAI. Using blood P4 profiles, median days to first insemination were 71, 76, and 96 for cows cycling at 21 d, 49 d, and anovulatory cows, respectively.

In our study, the median DIM at pregnancy results were 118 and 124 for ovulatory and anovulatory cows, respectively. Greater median DIM results for anovulatory cows were also reported in the two studies mentioned above. Here, the median DIM at pregnancy results were 126 and 156 for cyclic and anovulatory cows in herds with minimum hormonal interventions [17], and 103, 147, and 173 for cows cycling at 21 d, 49 d, and anovulatory cows, respectively [30].

The median DIM at first insemination results were 84 and 95 and the median DIM to pregnancy results were 121 and 143 for cows with and without an estrus event detected by an AAM system from 7 to 60 DIM. Cows with an estrus event from 7 to 60 DIM had an increased hazard of being inseminated by 200 DIM [HR = 1.80 (1.51–2.15); *p* < 0.001] and becoming pregnant by 300 DIM [HR = 1.84 (1.52–2.24); *p* < 0.001] compared to cows without an estrus event. This is in agreement with two previous studies from our group [1,3]. Cows without an estrus event within the VWP were both less likely to be inseminated by 100 DIM and become pregnant by 200 DIM. The median DIM at first insemination results were 70, 59, and 58 for cows with no, one, or multiple estrus events from 7 to 40 DIM [1], and 84, 74, and 74 for cows with no, one, or multiple estrus events from 7 to 60 DIM [3], respectively. The median DIM at pregnancy results were 127, 112, and 103 for cows with no, one, or multiple estrus events from 7 to 40 DIM [1], and 121, 96, and 92 for cows with no, one, or multiple estrus events from 7 to 60 DIM [3], respectively.

### 4.6. Targeted Reproductive Management

Targeted reproductive management (TRM) is a management approach that consists of identifying subgroups of cows that would benefit from specific interventions to optimize their reproductive performance. Once subgroups of cows with different reproductive potentials (i.e., probability to express estrus, probability of pregnancy at first postpartum AI) are identified, cows can be assigned to different TRM strategies. Anovulatory cows represent such a subgroup. However, anovulatory cows are not diagnosed on a routine basis, although it has been shown that an anovulatory condition until the end of the VWP has a strong negative effect on reproductive performance and consequently on the overall farm profitability [17,19,30]. According to our results, the AAM systems differed in their test characteristics (i.e., accuracy and sensitivity) to detect anovulatory cows from 7 to 60 DIM. Therefore, not all AAM systems can be used for TRM. Despite some misclassification of anovulatory cows, estrous expression from 7 to 60 DIM was associated with reproductive performance. Therefore, anestrus can be used as a risk factor for poor reproductive performance.

This approach is supported by two recent studies from our group [1,3]. Cows with at least 1 or ≥ 2 estrus events recorded by 40 [1] and 60 DIM [3] had greater odds of insemination before 100 DIM, received first AI earlier, and had a greater hazard of pregnancy up to 200 DIM. Moreover, cows with at least one estrus alert had a more intense estrus event at the first postpartum AI compared with cows with no recorded estrus event in early lactation. This has been the rationale for evaluating different intervention strategies in cows with and without an estrus event within the VWP [2,31]. Enrollment of cows without an estrus event within the VWP into a TAI protocol can help mitigate a delayed time to pregnancy. On the other hand, cows with estrus activity within the VWP should be inseminated based on estrus detection after the VWP as they have a greater hazard for estrus expression and greater overall fertility [2]. In a herd following TRM, false-positive cows (i.e., ovular within the VWP but not detected as such by the AAM system) would be enrolled into a TAI protocol although not necessary, and thus unnecessary costs for labor and hormones would be incurred. On the other hand, in false-negative cows (i.e., estrus within the VWP but anovular), one could expect a delayed time to pregnancy. This represents a missed opportunity.

### 4.7. Study Limitations

To the best of our knowledge, this is the first study comparing four AAM systems on their ability to detect anovulatory cows within the VWP. A limitation of our study is that the four AAM systems were not directly compared on the same farm or even all four AAM systems on the same cows. Different reproductive management strategies on the farms such as the definition of VWP must be considered. Generalizability to other settings and systems should be considered when applying these findings to different contexts. In a related study from Dolecheck et al. [6], five different AAM systems attached to 35 cows at the same time were tested for behavioral and physiological changes around estrus. However, here, the cows were enrolled at 56 DIM or later.

For HT, we had to exclude 32 cows due to missing or incomplete data. The reason for missing activity data remains speculative but it may have been due to malfunction of the AAM system or data transmission. This phenomenon has already been described earlier [1]. This clearly limits the practical use of this system for early identification of cows with poor predicted reproductive performance and to aid the decision-making process related to reproductive management.

While this study provides important insights, further research is warranted to validate and compare the performance of different AAM systems to detect anovulatory cows in a controlled setting. Future studies should involve multiple AAM systems installed on the same cows to obtain more accurate and comprehensive results.

## 5. Conclusions

The results of the present study provide further evidence that estrous expression within the VWP is a predictor of reproductive performance. Interestingly, estrous expression seems to have a higher predictive value than ovulation within the VWP based on the reproductive performance of the different subgroups. The AAM systems differed in their test characteristics to detect anovulatory cows from 7 to 60 DIM. Therefore, estrus alerts generated from AAM systems within the VWP cannot generally be used as a robust proxy for the resumption of cyclicity. Future studies should address the use of multiple AAM systems (intra-AAM system variation and between-AAM system variation) to detect anovulatory and anestrous cows within the VWP and the use of different intervention strategies in these cows to improve their reproductive performance. To compare multiple AAM systems regarding their performance on estrus detection, multiple AAM systems should be assigned to the same group of cows. Traits like estrus duration, estrus intensity as well as the number of estrus events, and the interval between estrus events within the VWP should be further evaluated regarding their suitability as predictors of fertility.

## Figures and Tables

**Table 1:** Descriptive information ^1^ of enrolled dairy farms.

			Farm	
Parameter	1	2	3	4
Average farm size (cows)	1003	639	669	615
Average 305 d milk yield (kg)	11,598	10,791	10,035	10,008
Milking frequency per day	2	3	2	2
AAM system ^2^	Smartbow	Heatime	Delpro activity meter	CowManager
AAM system software	Smartbow	DataFlow II	Delpro Farm Management	CowManager Control Center
Location of the AAM system	Ear tag	Neck mounted	Neck mounted	Ear tag
Average days in milk for a blood sample (P1/P2/P3) ^3^	(21/35/49)	(21/35/49)	(21/35/49)	(20/35/49)

^1^ Descriptive information of farms was obtained from the farm management software (herdeplus; dsp-Agrosoft GmbH, Ketzin, Germany). ^2^ All cows were equipped with automated activity monitor (AAM) systems for detection of estrus (farm 1: Smartbow, Zoetis Inc.; farm 2: Heatime, SCR Engineers Ltd.; farm 3: Delpro, AM 2, DeLaval; and farm 4: CowManager SensOor, Agis Automatisering) on the respective dairy farm. ^3^ The first blood progesterone sample (P1) was taken between 14 and 27 DIM, the second (P2) two weeks after the first sample, between 28 and 41 DIM, and the third sample (P3) two weeks after the second sample, between 42 and 55 DIM (±1 d).

**Table 2:** Descriptive information of enrolled dairy cows stratified by farm.

			Farm		
Parameter	1	2	3	4	Overall
Enrolled cows (*n*)	219	197	216	220	852
Parity (primiparous/multiparous)	51/168	72/157	57/159	53/167	221/631
Proportion (%) of primiparous cows	23.3	31.4	26.4	24.1	25.9
Anovulatory cows (%) ^1^	18.7	22.3	15.3	17.3	18.3
Anestrous cows (%) ^1^	13.7	26.9	61.6	10.5	28.1
Number of cows with BCS loss (*n*) ^2^	153	140	146	145	584
Number of cows with lameness (lame/not lame) ^3^	(45/174)	(42/155)	(48/168)	(40/180)	(175/677)

^1^ Cows were classified as (1) no estrus alert from 7 to 60 DIM by the respective AAM system (anestrus); (2) at least one estrus alert from 7 to 60 DIM by the respective AAM system (estrus). In addition, cows were classified as (1) none of the three blood progesterone concentrations exceeded 1.0 ng/mL (anovulatory); (2) at least one of the three blood progesterone concentrations was above 1.0 ng/mL (ovulatory). The first blood progesterone sample (P1) was taken between 14 and 27 DIM, the second (P2) two weeks after the first sample, between 28 and 41 DIM, and the third sample (P3) two weeks after the second sample, between 42 and 55 DIM (±1 d). ^2^ Body condition score (BCS) was scored at several points in time within the voluntary waiting period (first scoring between 14 and 28 days in milk, last scoring between 42 and 56 days in milk) on a 5-point scale (1 = thin to 5 = fat). Cows were defined with BCS loss when the BCS difference between the first and the last time of scoring (P1 vs. P3) had a negative value. ^3^ Lameness was scored on a 5-point scale. Cows were defined as lame when they were scored 3, 4, or 5 at one or more points in time of the three scorings (P1/P2/P3).

**Table 3:** Test characteristics for 4 automated activity monitoring systems [% (95% CI)] ^1^ to detect anovulatory cows within 60 DIM on the respective farms ^2^.

Sensor ^3^	Sensitivity	Specificity	NPV ^4^	PPV ^4^	Accuracy
SB (*n* = 219)	26.8 (14.2–42.9)	89.3 (83.3–93.5)	84.1 (78.1–89.0)	36.7 (19.9–56.1)	77.6 (71.5–83.0)
HT (*n* = 197)	63.6 (47.8–77.6)	83.6 (76.8–89.1)	88.9 (82.6–93.5)	52.8 (38.6–66.7)	79.2 (72.8–84.6)
DP (*n* = 216)	78.8 (61.1–91.0)	41.5 (34.3–49.0)	91.6 (83.4–96.5)	19.5 (13.2–27.3)	47.2 (40.4–54.1)
CM (*n* = 220)	23.7 (11.4–40.2)	92.7 (87.4–95.7)	85.3 (79.6–89.9)	39.1 (19.7–61.5)	80.5 (74.6–85.5)

^1^ Cows (*n* = 852) were equipped with automated activity monitoring (AAM) systems (SB: Smartbow, Zoetis Inc.; HT: Heatime, SCR Engineers Ltd.; DP: Delpro, AM 2, DeLaval; and CM: CowManager SensOor, Agis Automatisering). ^2^ Each AAM system (SB, HT, DP, and CM) was used on a different dairy farm. ^3^ Cows were classified as (1) no estrus alert from 7 to 60 DIM by the respective AAM system (anestrus); (2) at least one estrus alert from 7 to 60 DIM by the respective AAM system (estrus). In addition, cows were classified as (1) none of the three blood progesterone concentrations exceeded 1.0 ng/mL (anovulatory); (2) at least one of the three blood progesterone concentrations was above 1.0 ng/mL (ovulatory). The first blood progesterone sample (P1) was taken between 14 and 27 DIM, the second (P2) two weeks after the first sample, between 28 and 42 DIM, and the third sample (P3) two weeks after the second sample, between 43 and 56 DIM. ^4^ NPV = negative predictive value; PPV = positive predictive value.

**Table 4:** Proportion (%) of cows categorized based on blood progesterone concentration and the automated activity monitoring (AAM) ^1^ system from 7 to 60 DIM on the respective farms ^2^.

Category	SB (*n*)	HT (*n*)	DP (*n*)	CM (*n*)
Estrus	86.3 (189)	73.1 (144)	38.4 (83)	89.6 (197)
Ovulation	72.6 (159)	65.0 (128)	35.2 (76)	76.4 (168)
Anovulation	13.7 (30)	8.1 (16)	3.2 (7)	13.2 (29)
No estrus	13.7 (30)	26.9 (53)	61.6 (133)	10.5 (23)
Ovulation	8.7 (19)	12.7 (25)	49.6 (107)	6.4 (14)
No ovulation	5.0 (11)	14.2 (28)	12.0 (26)	4.1 (9)

^1^ All cows were equipped with automated activity monitoring (AAM) systems for the detection of estrus (SB: Smartbow, Zoetis Inc.; HT: Heatime, SCR Engineers Ltd.; DP: Delpro, AM 2, DeLaval; and CM: CowManager SensOor, Agis Automatisering). ^2^ Farm 1 (*n* = 219 cows) = SB; farm 2 (*n* = 197 cows) = HT; farm 3 (*n* = 216 cows) = DP; and farm 4 (*n* = 220 cows) = CM.

## Data Availability

The data are contained within the article. The raw data are available upon request from the corresponding author.
